# Changes and Analytical Techniques in Volatile Flavor Compounds in Dried Agricultural Products: A Review

**DOI:** 10.3390/foods14203531

**Published:** 2025-10-16

**Authors:** Pengxiao Chen, Siyuan Zhao, Chengyu Li, Tingting Zhang, Yanjia Xing, Kai Zhang, Jiale Lv, Wenxue Zhu

**Affiliations:** 1 School of Food and Strategic Reserves, Henan University of Technology, No. 100 Lianhua Street, Zhengzhou 450001, China; cpx2020@haut.edu.cn (P.C.); zhaosiyuan1124@126.com (S.Z.); zttwssy@163.com (T.Z.); 18555952353@163.com (Y.X.); 15672856373@163.com (K.Z.); ljl99966@126.com (J.L.); 2 School of International Education, Henan University of Technology, No. 100 Lianhua Street, Zhengzhou 450001, China; 18737077176@163.com

**Keywords:** drying, agricultural products, volatile flavor compounds (VOCs), reactions, techniques

## Abstract

Drying is a key step in the primary processing of agricultural products. It alters the type and content of volatile flavor compounds (VOCs), imparting distinctive flavors to the products. This article reviews the common physical and chemical reactions occurring during the drying of agricultural products, the types of VOCs, the detection and analysis methods, and the research progress on the effects of different drying methods on the VOCs of agricultural products. The article serves as a reference point for further research into the VOCs of agricultural products after drying, and provides a theoretical foundation for subsequent research into the development and utilization of agricultural resources.

## 1. Introduction

Agricultural products refer to plants, animals, and microorganisms obtained through agricultural production activities, as well as the primary processed goods derived from them. These products primarily include grains, vegetables, fruits, livestock and poultry, aquatic products, and forest products. They are vital sources of food and industrial raw materials for human survival. These products typically retain their natural attributes and undergo minimal processing, exhibiting distinct seasonal and regional characteristics. Drying represents a pivotal primary processing method for agricultural products. The physical and chemical reactions that occur during the process of drying have been shown to alter the type and content of VOCs. This process is instrumental in shaping the unique flavour profile. The flavour of agricultural products is a significant factor in the evaluation of food quality. The distinctive flavour of durian is attributable to a chemical interaction between esters and sulfur-containing compounds. These contribute to a fruity aroma and a characteristic pungent durian odor [1]; The characteristic flavor of bananas is attributed to isoamyl acetate and isobutyl acetate [2]. The most prevalent drying techniques employed in the agricultural sector are hot air drying and natural drying. While hot air drying can rapidly reduce the moisture content of agricultural products, it also results in significant degradation of VOCs. Natural drying can, to some extent, better retain the VOCs; however, the longer drying times are not well suited to industrial production. In recent years, in order to maintain the optimal flavour after drying, freeze drying, microwave drying and infrared drying, and other modern drying technologies have gradually become employed in the drying process [3].

**Figure 1:**
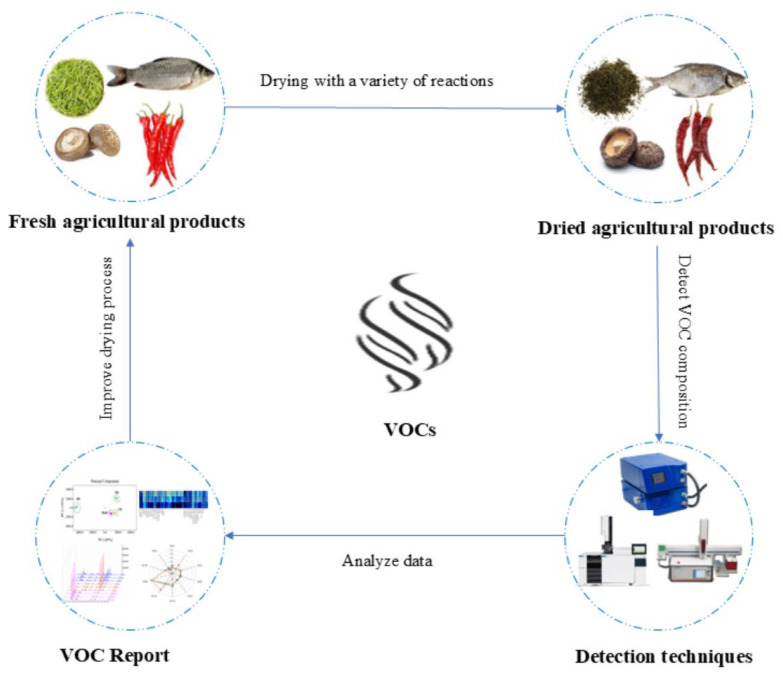
The changes and analytical techniques of VOCs in dried agricultural products.

The drying process of agricultural products is characterised by a range of physical and chemical reactions. These reactions interact with each other, thereby imparting a distinctive flavour profile to the final product. The most common VOCs found in agricultural products are aldehydes, esters, ketones, and alcohols. Zhou et al. [4] found that charcoal roasting of *Lentinus edodes* facilitates conversion of sulfur compounds while increasing alcohols and aldehydes, reducing volatile substances but enhancing umami flavor. The VOCs are identified through an electronic nose, GC-MS, and GC-IMS. The analysis of VOCs is conducted through the use of Descriptive Sensory Analysis (SA) and the odor activity value (OAV). The effects of different drying methods on the VOCs are subject to variation. In conclusion, the changes and analytical techniques of VOCs in agricultural products following the drying process are illustrated in Figure 1.

A review of recent literature on VOCs in dried products reveals a predominant focus on a particular drying method or a specific agricultural product. Zhang et al. [5] reviewed the development of flavor during drying and applications of edible mushrooms. Okonkwo et al. [6] reviewed the Changes in flavor profile of vegetable seasonings by innovative drying technologies. Wang et al. [7] reviewed thermochemical reactions in tea drying shape the flavor of tea. The drying of agricultural products exerts a complex influence on VOCs. By regulating the drying process and maintaining equilibrium among VOCs, it is possible to produce agricultural products that meet desired flavor specifications. The objective of this review is to: (1) elucidate the flavor-related physical and chemical reactions that occur during the drying of agricultural products. (2) introduce the technologies and analytical methods that are currently available for the detection of VOCs. (3) elucidate the effect of different drying methods on VOCs in agricultural products. The review offers a theoretical foundation for research on the flavor of dried agricultural products and the development and utilization of agricultural resources.

A comprehensive search was conducted using keywords such as “drying”, “agricultural products”, “volatile flavor compounds”, “Maillard reaction”, “electronic nose”, and “gas chromatography-mass spectrometry”. This search yielded over 400 articles, which were then reviewed using the Web of Science, PubMed, and ScienceDirect databases. The present study has yielded insights into the flavor profiles of agricultural products, the various reactions that occur during the drying process, and the characteristics of different drying methods. The findings presented herein facilitate the completion of this paper.

## 2. Reactions Associated with VOCs During Drying

Agricultural products are a rich source of proteins, fats, carbohydrates, and other organic substances. The internal substances of agricultural products undergo a series of physical and chemical reactions during the drying process, which significantly affect the VOCs of agricultural products.

### 2.1. Maillard Reaction

**Figure 2:**
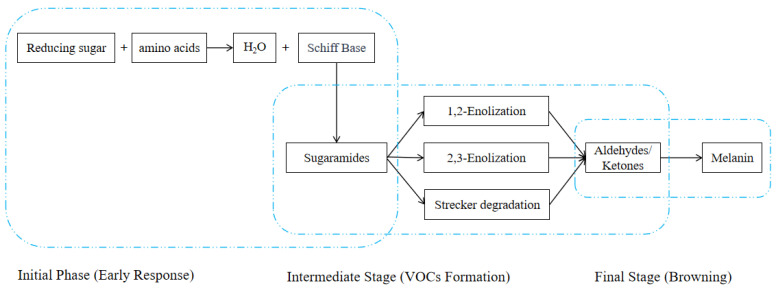
Maillard Reaction Process.

The Maillard reaction, also known as non-enzymatic browning, is a series of chemical reactions that occur between reducing sugars and proteins [8,9]. The Maillard reaction, a pivotal browning reaction in the domain of food processing, is initiated by the interaction of amino acids and reducing sugars when exposed to elevated temperatures [10]. As shown in Figure 2, the reaction is comprised of three distinct stages: Initially, the amino group and the sugar’s carbonyl group condense to form Schiff bases, which undergo Amadori rearrangement to create sugaramides. During the intermediate stage, sugaramides undergo a series of chemical reactions that result in the formation of flavor precursors, including aldehydes, ketones, and reducing ketones. Strecker degradation produces characteristic aldehydes while concurrently forming nitrogen-containing heterocyclic compounds (e.g., pyrazines, furans). In the final stage, the condensation and polymerization of small-molecule products result in the formation of melanoidins, which contribute to the brown coloration and complex aromas characteristic of food products [11,12]. This reaction necessitates elevated temperatures and a neutral to alkaline environment, rendering it a prevalent process in the food industry [13]. The minimum temperature at which the Maillard reaction occurs is 60 °C, which is the same as the common drying temperature employed for agricultural products. The majority of agricultural products are rich in sugars and proteins, which makes the Maillard reaction the most significant in the drying process of agricultural products. Wu et al. [14] found that the thermal degradation of carbohydrates, lipids, amino acids, and Maillard reactions resulted in the generation of hydrocarbons and alcohols in both samples as the drying process of seaweeds (*Bangia fusco-purpurea*) progressed. Liu et al. [15] conducted a study on the VOCs of jujube fruit (*Ziziphus jujuba* Mill.) after dehydration. They found that 2-hexenal, 1-nonanal, oct-3-enal, benzaldehyde, oct-1-en-3-ol, and 2-pentylfuran were the VOCs with an OAV greater than 1. The key to controlling dried jujube aroma is the oxidation reaction, the Maillard reaction of fatty acids, as well as amino acids and monosaccharides. The Maillard reaction process is a complex and richly volatile flavor formation process. The products of it are primarily aldehydes, but also include ketones, alcohols, esters, aromatics, alkanes, and heterocyclic compounds. Among the compounds above, heterocyclic compounds (including pyrazines, pyrroles, pyridines, thiophenes, thiazoles, and furans) exhibit distinctive flavor profiles, with the majority displaying roasted characteristics. It is established that these compounds impart distinctive flavor profiles to agricultural products [16].

### 2.2. Lipid Oxidation Reactions

**Figure 3:**
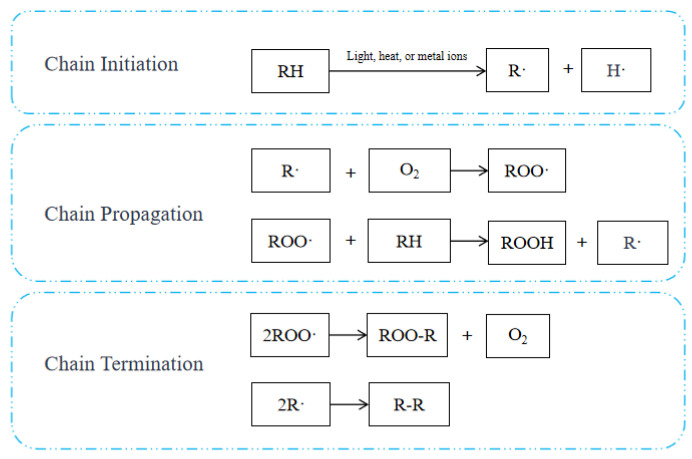
Auto-oxidation Free Radical Chain Reaction.

Lipid oxidation can be classified into three categories: auto-oxidation, enzymatic oxidation, and photosensitive oxidation [17,18,19]. The principal forms of oxidation that occur in produce during the drying process are auto-oxidation and enzymatic oxidation. Auto-oxidation is the primary pathway for lipid oxidation, involving a free radical chain reaction, as shown in Figure 3. The chain initiation phase is the first stage of the process. The abstraction of methylene hydrogen from unsaturated fatty acids, as catalyzed by light, heat, or metal ions, results in the formation of lipid radicals. The subsequent phase is referred to as the “chain propagation phase”. Lipid radicals react with oxygen to generate peroxy radicals, which then abstract hydrogen from other lipids. This process produces hydrogen peroxides and new lipid radicals in a cyclic reaction. The chain termination phase is the final stage of the process. Termination is defined as the cessation of propagation, which can occur via two primary mechanisms. One such process is the reaction between two radicals that leads to the formation of nonradical products. The alternative mechanism involves a reaction with an antioxidant, leading to the formation of a more stable and less reactive radical. The decomposition of hydrogen peroxide results in the formation of rancidity-inducing substances, including aldehydes and ketones [20,21]. In their study of the natural drying of traditional Tibetan yak jerky, Han et al. [22] observed that as the moisture content decreased, the protein and fat content increased, while the ratio of monounsaturated to polyunsaturated fatty acids decreased. The elevated altitude accelerated the oxidation of lipids and promoted the production of VOCs. In their research, Zhao et al. [23] examined the drying process of Pacific saury (*Cololabis saira*) and discovered that UV-assisted cold-air drying can effectively regulate the extent of oxidation in Pacific saury oils. Furthermore, this approach resulted in the generation of Pacific saury with a heightened content of methyl butyrate, propyl caproate and butyl butyrate, which has the potential to improve the flavor of saury. Kaban et al. [24] identified a range of volatile compounds in salted-dried goose, including aldehydes, aliphatic and aromatic hydrocarbons, esters, alcohols, terpenes, ketones, sulfur compounds, and furans. Some of these compounds were found to be formed as a result of lipid oxidation. Hu et al. [25] revealed that unsaturated fatty acid oxidation during coffee peel drying produces E-2-decen-1-ol and furaneol, primary sources of the fruity and caramel aromas in coffee peel. Autooxidation is the primary type of lipid oxidation, representing a spontaneous, unsaturated fatty acid-to-oxygen reaction. Enzymatic oxidation is the oxidation of unsaturated fatty acids catalyzed by fat-oxidizing enzymes. The primary flavor substances produced by lipid oxidation reactions are aldehydes and alcohols, with a higher percentage of aldehydes. Agricultural products such as meat and oilseed crops are rich in lipids, and lipid oxidation reactions often occur during drying. Controlled oxidation of lipids within specified limits is conducive to the formation of special flavors in dried agricultural products.

### 2.3. Enzymatic Reactions

Agricultural products are rich in a variety of endogenous enzymes, including polyphenol oxidase, fat oxidase, and peroxidase, among others. Enzymatic reactions can alter the intrinsic flavor profile of produce. Chen et al. [26] identified sulfur flavor as the dominant flavor compound in dried shiitake mushrooms, resulting from enzyme-produced heterocycles in lentinic acid during drying. In their study on the hot air drying of mango, Mukhtar et al. [27] found that both the drying temperature and air velocity had an effect on the catalase and polyphenol oxidase activities in mango. Drying temperature had a more pronounced effect on the degradation rate of catalase. The study by Hu et al. [28] revealed a decline in the activities of three pivotal enzymes (LOX, ADH, and AAT) of lemons in samples subjected to three drying techniques: integrated freeze drying, conventional freeze drying, and hot-air drying. The interconversion between these enzymes, fatty acids, and a range of volatiles was found to be intimately linked to the formation of volatile compounds. Enzymes are biological catalysts that accelerate reactions by facilitating the rapid interaction of specific substances within Agricultural Products. This process facilitates the production of VOCs during the drying phase.

### 2.4. Fermentation Reactions

The fermentation reaction is a process whereby organic matter in agricultural products is transformed, resulting in the production of volatile components that impart a distinctive flavor [29,30]. The process of fermentation is driven by the action of microorganisms that catalyze the breakdown of organic compounds, such as glucose, into smaller metabolic products through a series of enzymatic reactions. Concurrently, these microorganisms release energy. The core processes encompass glycolysis, which involves the conversion of glucose into pyruvate, and the subsequent conversion of pyruvate into products such as ethanol and lactic acid under the influence of various microorganisms. The drying of agricultural products in the presence of select microorganisms may give rise to a microbial fermentation reaction when conducted under suitable environmental conditions. The fermentation reaction does not necessitate a high temperature. For example, the fermentation of bread is frequently conducted at 28 to 30 °C, while the fermentation of spicy cabbage is often carried out at 15 °C. Lee et al. [31] discovered that the amount of VOCs was markedly elevated in dry-aged beef in comparison to wet-aged beef. Microbial metabolism serves as a primary contributor to this phenomenon, resulting in the production of 2-methylbutanal, 2-methylpropanal and 1-butanamine. Juhari et al. [32] demonstrated that roselle (*Hibiscus sabdariffa* L.) that were sun-dried on cloudy days were incompletely dried and thus fermented to a certain extent. This led to a substantial increase in the concentration of specific ketones (2-heptanone, 2-octanone, and 2-undecanone), esters (hexyl acetate, phenethyl acetate, and methyl hexanoate), and alcohols (pentanol and phenethyl alcohol). Li et al. [33] conducted research that demonstrated substantially higher concentrations of alcohol, primarily ethanol, in wet-aged beef loin than in wet-aged beef. Additionally, there was a substantial elevation in ethanol concentration over time, which is presumed to be the consequence of lactic acid bacteria fermentation. Because of their involvement in the curing process of agricultural products, microorganisms facilitate the production of VOCs in the resulting product, thereby imparting it with a distinctive flavor.

### 2.5. Additional Reactions

Moreover, the drying process is associated with a range of other physical and chemical reactions. Esters, the products of esterification, have pleasant aromas, such as ethyl hexanoate with a fruity aroma, ethyl caprylate with a wine-like flavor, and ethyl decanoate with a coconut aroma. High temperature accelerates the process of esterification reaction; adding wine and vinegar when cooking fish, the alcohols in the wine and the acids in the vinegar will generate esters under the effect of heat to enhance the flavor of the fish. In their study of the drying of Qingzhuan tea, Fang et al. [34] identified the degradation of carotenoids and the methylation of gallic acid as key reactions in the production of VOCs following the drying process. The impact of drying procedures on VOCs of golden pompano (*Trachinotus ovatus*) was investigated by Chen et al. [35]. The results indicated that the myofibrillar proteins in golden pompano subjected to hot air drying and heat pump drying underwent substantial oxidation and degradation.

## 3. VOCs Changes During Drying

The combination of physical and chemical reactions involved in the drying of agricultural products results in alterations to the types and concentrations of VOCs, consequently influencing the flavor profile of the products in question. Rajkumar et al. [36] discovered the presence of novel compounds, including 3-methyl furan, hexanol and terpinyl acetate, in dried tomatoes that were not observed in fresh tomatoes. Furthermore, the drying process diminished the off-odour characteristics of the produce, thereby enhancing its economic value. Bi et al. [37] discovered that fresh rape bee pollen exhibited a pronounced odor of 2-methylbutyric acid, and the concentration of this acid was markedly diminished following a drying process, thereby improving the rape bee pollen’s flavour quality. The flavor of agricultural products perceived by the human sense of smell is determined by the combination of the content and threshold of the VOCs present. Following drying, aldehydes and esters are present in significant quantities and possess a comparatively low threshold, which makes them the main taste-presenting volatile flavors in agricultural products. Table 1 presents a selection of the key VOCs found in dried agricultural products; the key VOCs vary among different agricultural products. For the majority of agricultural products, aldehydes are the predominant flavor compounds.

### 3.1. Aldehydes

Aldehydes contribute significantly to the scent of agricultural products and have a low threshold for aroma. The existence of minute quantities of aldehydes can impart a mellow aroma to agricultural products, whereas excessive aldehyde levels can result in the production of an irritating odor. Benzaldehyde has an almondy, fruity flavor, and phenylacetaldehyde has a hyacinth-like aroma, and a sweet, fruity flavor when diluted. The discrepancies in aldehydes between products resulting from disparate drying techniques are predominantly ascribable to lipid degradation. Additionally, aldehydes can be generated by Strecker oxidation and unsaturated fatty acid oxidation. It has been noted that higher temperatures promote the decomposition of aldehydes. Yang et al. [38] discovered that the maximum amount of aldehydes was present in *Lyophyllum* decastes following the drying process. The approach that produced the highest aldehyde content was hot air drying, reaching 32.87%. This was followed by vacuum freeze-drying and hot air combined with vacuum drying. Additionally, vacuum freeze drying resulted in a notable elevation in the concentration of octanal and heptanal, which have a fruity aroma. In their study of mango drying, Fratianni et al. [39] identified the presence of aldehydes, including hexanal, heptanal, trans-2-nonenal and 2-6-nonadienal, in the dried mango (*Mangifera indica* L.). These compounds were produced through a range of physical and chemical reactions, including those involving fatty acids. In a study conducted by Sung et al. [40], the drying process of Gongliao Gelidium seaweed was analyzed. The results showed that the unpleasant hexanal molecule was eliminated by washing and sun drying, while concurrently enhancing long-chain unsaturated alkylaldehydes. These alterations resulted in the production of an odor profile characterized by green, waxy, and floral odors. In their study of charcoal-roasted mutton, Liu et al. [41] observed that the major aroma components in roasted lamb increased gradually in the course of roasting, reaching a maximum at 10 min. Of these compounds, hexanal was identified as the most significant, exhibiting the highest concentration, OAV, and contribution. Aldehydes are present in considerable quantities and in a variety of forms in dried produce, imparting a distinctive flavor profile to the product.

### 3.2. Ketones

The generation of ketones is primarily facilitated by the process of lipid oxidation, which occurs through the application of heat. The aroma of short-chain ketones is described as burnt and fatty, while that of long-chain ketones is perceived as floral. In a study of spray drying of yak milk powder, Feng et al. [42] noted an elevation in aldehydes and ketones in yak milk upon contact with hot air during the drying process. The ketones were found to be a major contributor to the overall flavor profile of the yak milk powder, exhibiting characteristics associated with milk, fat, and fruit. In their study of *Morchella sextelata* drying, Li et al. [43] found that natural air drying was an inefficient method, resulting in the loss of flavor substances. Conversely, hot air drying was observed to promote the production of heterocyclic and ketone VOCs in *Morchella sextelata*, imparting a distinctive roasted flavor. The thermal drying method has been demonstrated to facilitate the production of ketone VOCs in produce, thereby imparting a distinctive flavor profile.

### 3.3. Esters

The free fatty acids generated by lipid oxidation during the drying of produce combine with alcohols to form esters. Among these, the short-chain esters are typically more volatile and can impart fruity and sweet flavors to the produce. In an examination conducted by Yao et al. [44], the effects of various drying techniques on sweet corn were investigated. The researchers discovered that the 2-(trimethylsilyl)-oxy)-trimethylsilyl ester content ranged from 7.72% to 4.47% when different drying techniques were employed. However, butyl ester and 5-butyldihydro-2(3H)-furanone were only identified in fresh material. All four drying treatments (vacuum freeze-drying, hot-air drying, microwave drying, and infrared drying) resulted in a reduction of these esters. In a study of strawberry drying conducted by Abouelenein et al. [45], it was determined that the ester content of strawberries subjected to microwave drying, hot air drying, freeze drying, and shade air-drying treatments was, in order, 0.35%, 17.48%, 24.05%, and 34.34%, respectively. The ester content of the fresh strawberries was 36.96%. Khatun et al. [46] studied cricket drying and found that freeze-dried crickets had the highest content of ester volatiles, which was much higher than crickets dried using oven drying and rinsing drying. The content of VOCs in agricultural products is subject to significant variation depending on the drying method employed, which in turn gives rise to distinctive flavor profiles in the finished product.

### 3.4. Alcohols

Alcohols are primarily derived from the oxidative decomposition of fats and the reduction of carbonyl compounds. Alcohols possess a higher flavor threshold than other chemical classes. The flavour profiles of agricultural products are barely affected by straight-chain saturated alcohols. However, as the carbon chain lengthens, these alcohols can impart flavors that are higher in intensity, such as fatty, light, and woody, respectively. In contrast, unsaturated alcohols exhibit lower flavor thresholds and contribute more significantly to overall flavor perception. The research conducted by Zhang et al. [47] investigated the influence of varying drying AC voltages on goji berries (*Lycium barbarum* L.), revealing that alcohols and aldehydes constituted the most predominant flavor compounds in the dried goji berries. Xu et al. [48] discovered that the alcohol content of *Pingyin roses* following vacuum freeze-drying exceeded 400 mg/kg, imparting a floral and fruity flavor to the dried roses. Among the identified compounds, 3,7,11-trimethyl-2,6,10-decatrien-1-ol is a distinctive polyunsaturated alcohol present in roses, exerting a pronounced influence on the floral and fruity flavors of the roses. In their study of the dehydration process of Rosa rugosa flower, Qiu et al. [49] observed that both fresh and dried samples exhibited a markedly elevated concentration of alcoholic volatile flavors. The highest concentration was observed in (R)-3,7-dimethyl-6-octen-1-ol, which is one of the most characteristic flavors associated with Rosa rugosa flowers. Alcohols represent a significant class of VOCs that exert a marked influence on the flavor of produce following the drying process.

**Table 1:** Key volatile compounds.

No.	Agricultural Products	Key Volatile Compounds
1	Chili pepper (*Capsicum annuum* L.) [50]	(E)-2-nonanal, 3-methylbutanal-M, 2-methylbutanal-M
2	*Dendrocalamus brandisii* shoots [51]	e 2-Nonenal, 3-Methylbutanal, trans-2,cis-6-Nonadienal
3	*Rosmarinus officinalis* L. [52]	Camphor, α-pinene, and α-terpineol
4	Turnip (*Brassica rapa* L.) [53]	2-azido-2,3,3-trimethyl-butane and hexanal
5	*Zanthoxylum bungeanum* Leaves [54]	Linalool and α-terpinyl
6	Persimmon (*Diospyros kaki* L.) [55]	Decanal
7	Eel (*Muraenesox cinereus*) [56]	Hexanal, nonanal, benzaldehyde, octanal, and oct-1-en-3-ol
8	Thyme (*Thymus vulgaris* L.) [57]	Thymol, carvacrol
9	Beef loin [58]	Toluene, 1-Butanol
10	Ginger (*Zingiber officinale Roscoe*) [59]	Dihydro-α-curcumene
11	Walnut (*Juglans regia* L.) [60]	(E,E)-2,4-decadienal, oct-1-en-3-ol and pyrazines
12	Tree tomato [61]	Eucalyptol

## 4. Techniques for the Detection of VOCs

The application of VOC detection technology in the drying process allows for the comparison of changes in the types and levels of VOCs before and after drying. It makes it possible to get comprehensive information on how the drying operation affects the VOCs of agricultural products, which can then be employed to further optimize the drying process.

### 4.1. Electronic Nose

The electronic nose (E-nose) is a bionic instrument that employs electrochemical sensing arrays in conjunction with pattern recognition techniques to rapidly identify and differentiate between various types of odors, thereby forming a spectrum of the sensor array’s response to that odor [62]. The electronic nose has several advantages, including fast detection, low cost, simple operation, and strong objectivity, and is therefore widely used in food flavor research [63]. Table 2 shows the Sensor array and its main properties of E-nose; each array is indicative of the content of a specific compound, and the radar chart in its entirety is capable of characterizing the flavor profile of agricultural products. In a study, Bu et al. [64] employed the electronic nose technique to investigate the impact of drying red sea bream surimi powder on VOC. The results demonstrated that alcohols, aldehydes, and ketones exhibited the most pronounced increase, as illustrated by the radar diagram of the E-nose, implying that the drying procedure can cause the VOC concentration to rise. By employing the electronic nose technique, Han et al. [65] examined the vacuum freeze-drying of leeks and discovered that the highest concentrations of organic and inorganic sulfides were present in the dried leeks. The application of electronic nose technology has become a prevalent methodology for the characterization of VOCs in agricultural products following the drying process.

### 4.2. Gas Chromatography

Gas chromatography (GC) is a technique that can be used to separate complex gas components and qualitatively analyze unknown compounds. It does this by measuring the time taken for a component to elute from the column, and it is capable of providing high-speed analysis and high separation efficiency. Headspace Solid Phase Microextraction (HS-SPME) can often be used in combination with gas chromatography for the analysis of VOCs. HS-SPME is a technique whereby the sample to be tested is placed in a headspace vial. The volatile components are then volatilized from the sample matrix by heating, and the gas is extracted for chromatographic analysis. Thus, the composition and content of the VOCs in the sample can be investigated [66]. During the extraction process, the fiber head is exposed to the sample’s headspace. The extracting fiber head, coated with an adsorbent, then enriches the volatiles distributed in the headspace of the sample, thus completing the extraction.

#### 4.2.1. Gas Chromatography–Mass Spectrometry

The application of gas chromatography–mass spectrometry (GC-MS) technology enables the acquisition of a total ion flow chromatogram in conjunction with the mass spectral data associated with each peak. Subsequently, the data can be cross-referenced with a standard mass spectral library, thereby facilitating the identification of the structural composition of volatile substances present in each peak. Furthermore, the relative abundance of each component can be determined through the application of peak area normalization methods. The total ion chromatogram was obtained through the GC-MS technique for the purpose of detecting the drying process of *Tricholoma matsutake* [67]. Sun et al. [68] employed the GC-MS technique to examine the alterations in VOC in garlic following drying. Their findings revealed that the type of sulfur-containing compounds in fresh, infrared dried, and vacuum microwave dried garlic was 14, 8, and 7, respectively. Additionally, the types and quantities of sulfur-containing compounds in infrared-dried and microwave vacuum-dried garlic were found to be highly similar. Shi et al. [69] employed the GC-MS technique to investigate dried star anise (*Illicium verum* Hook.f.) and identified 101 VOC in it. They belonged to phenols, aldehydes, acids, ketones, ethers, alkanes, alcohols, and esters. The number of compounds was 2, 8, 6, 4, 3, 9, 10, 18, 40, and 1, respectively. The olefins, esters, alcohols, and ethers were identified as the primary aroma components. Zhu et al. [70] employed GC-MS to examine the volatile compounds present in blanching treatments combined with commercial drying methods for Irish brown seaweed (*Alaria esculenta*). The analysis revealed the presence of over 76 distinct VOCs, with aldehydes being the most prevalent. GC-MS is a suitable method for identifying VOCs in agricultural products after drying.

#### 4.2.2. Gas Chromatography–Ion Mobility Spectrometry

Given the disparate detection principles at play, the types and contents of VOCs identified by GC-MS and GC-IMS techniques for the same sample may vary [71]. Gas chromatography–ion mobility spectrometry (GC-IMS) is an analytical technique that is employed for the detection of volatile compounds in a variety of matrices. The technique is based on the differential migration speeds of distinct gas-phase ions in an electric field, which enables the analysis of compounds with high separation and low detection limits. Moreover, it is able to detect aroma substances with low concentration but high relative odor intensity, which GC-MS may be unable to accomplish. The fingerprint of the products can be obtained by GC-IMS technique, the fingerprint of Schisandra chinensis VOCs by different drying methods [72]. By comparing the fingerprints, it is possible to distinguish the composition of compounds in different samples, which can be employed to facilitate the identification of VOCs in agricultural products after drying. Furthermore, comparisons may elucidate the distinctions in aroma between different drying methods. In their study of the VOCs present in chili peppers following the hot air drying, Get et al. [73] employed the use of GC-IMS. Their findings revealed the presence of 45 distinct VOCs, including 11 esters, 11 aldehydes, 9 alcohols, 5 ketones, 3 furans, 3 acids, 2 pyrazines, and 1 ether. Additionally, they observed a decline in the aldehyde and alcohol content as the drying process progressed, accompanied by an initial increase and subsequent decrease in ester content. A recent study by Li et al. [74] examined VOCs in Chinese dry-cured hams. GC×GC-ToF-MS and GC-IMS identified 265 and 45 VOCs in 6 Chinese dry-cured hams. The results demonstrated that the GC-IMS exhibited comparable clustering patterns on both the PCA and MFA plots, suggesting that this method is an effective approach for the rapid identification of VOCs in dry-cured hams. In a study of hot-air drying of citrus fruit peels, Wang et al. [75] identified a total of 29 VOCs using GC-IMS. The drying process resulted in a reduction in the concentrations of linalool oxide, benzaldehyde, (E)-2-heptenal, and 1-pentanol, while the concentrations of pentanal, decanal, linalool, and (E)-2-hexenol increased. GC-IMS is a suitable method for identifying VOCs in agricultural products after drying.

The detection of volatile constituents of agricultural products after drying can be achieved through the use of various analytical techniques, including E-nose, GC-MS, and GC-IMS. E-nose is a rapid and cost-effective method that is capable of identifying overall flavor differences between samples. In contrast, GC-MS and GC-IMS exhibit differing sensitivities to different types of substances, making them suitable for specific applications. In practice, these techniques are often employed in conjunction to ensure the comprehensive retention of information pertaining to the volatile components of agricultural products. Rong et al. [76] investigated the volatiles of Pu-erh tea with different storage years, optimized the retention of comprehensive information inherent to Pu-erh tea volatile constituents through the integration of multiple detection techniques, and employed diverse statistical analyses to rapidly distinguish Pu-erh teas with distinct storage periods.

## 5. Analytical Methods of VOCs

Following the acquisition of data pertaining to volatile flavour compounds in desiccated agricultural products, a variety of analytical methodologies may be utilised for the purpose of data analysis. These methods integrate substantial amounts of odour and flavour data, thereby assisting researchers in perceiving the overall characteristics of volatile flavour substances following the drying process. Common approaches to the analysis of flavour properties include Descriptive Sensory Analysis and Odor Activity Value.

### 5.1. Descriptive Sensory Analysis

Descriptive Sensory Analysis (SA) of flavor employs established sensory evaluation techniques, conducted by duly trained sensory analysts. To evaluate the quality of food products, these experts use the senses of sight, smell, taste, touch, and hearing [77]. The olfactory component of food sensory evaluation can be employed for the investigation of VOCs in dried agricultural products. Zhu et al. [78] employed the flavor sensory evaluation technique to investigate the alterations in VOCs subsequent to horseradish drying. Their findings indicated that the scoring values for natural drying, hot air drying, vacuum drying, vacuum freeze drying, and vacuum freeze drying were, in descending order, 1, 2, 3, 4, and 5, respectively. Yang et al. [79] employed flavor sensory evaluation techniques to investigate the green tea. The results demonstrated that green tea dried for 30 min exhibited the highest flavor sensory evaluation score, while tea dried for 5 min demonstrated the lowest. With regard to aroma, green tea displayed a faint scent for 5–15 min, followed by a chestnut-like aroma for 20–35 min, and finally a high fire fragrance for 40–45 min.

### 5.2. Odor Activity Value

The Odor Activity Value (OAV) is defined as the ratio of the mass concentration of a VOCs to its aroma threshold. It is used to assess the degree of contribution of the VOCs to the sample’s aroma. In essence, the larger the OAV, the greater the contribution of the ingredient to the overall flavor of the sample. The calculation is shown in Equation (1):

OAV = C_i_/OT_i_ (1)

OAV represents the Odor Activity Value; C_i_ represents the percentage of VOCs, %; The OT_i_ represents the threshold of a VOC that can be perceived by the human senses, μg/kg. An OAV higher than 1 implies that the volatile flavouring component in question contributes more to the overall aroma of the sample. An OAV between 0.1 and 1 suggests that the VOCs change the overall aroma of the sample. An OAV below 0.1 shows that the VOCs have no practical influence over the overall aroma of the sample.

In their study of dried Citrus reticulata Ponkan and Chachi peels, Yu et al. [80] observed that the OAV of 2-methoxy-4-vinylphenol and linalool was higher in dried Ponkan peels, while the OAV of methyl anthranilate, methyl methanthranilate, perillal, α-sinensal, γ-terpinene, and terpinolene was higher in Chachi peels. The two citrus peels could be distinguished on the basis of their respective VOCs. The study by Liu et al. [81] examined the drying processes of gonggan and indicated that the freeze-drying method resulted in the highest OAV of (E,E)-2,4-decadienal. In comparison, the hot-air drying process at 60 °C yielded the highest OAV of (E)-2-nonenal, while the 70 °C and 80 °C treatments resulted in the highest OAV of (E)-2-nonenal. In their investigation, Lai et al. [82] identified a number of VOCs resulting from the drying of *Hypsizygus marmoreus*. These included trimethylamine, 3-octanone, (E)-2-octenal, and dimethyl disulfide, which were identified as contributing to the seafood aroma. The order of their OAV was as follows: heated freeze-drying > unheated freeze-drying > heat-pump drying > hot air drying. The OAV is a mathematical formula that allows the quantification and comparison of the effect of a VOCs on overall flavor. It does so by introducing a quantitative measure of the substance’s impact on the overall flavor profile.

## 6. The Impact of Drying Methods on the VOCs

The diverse range of drying techniques employed in the processing of agricultural products utilize varying mechanisms to induce specific alterations in the VOCs present in these products [5]. It would be beneficial to consider the defining characteristics and the overall impact of agricultural products, as well as the available drying methods, in order to identify the most appropriate method.

### 6.1. Hot Air Drying

The primary factor influencing the efficacy of hot air drying is temperature. As temperature increases, the intensity of the internal Maillard and lipid oxidation reactions in agricultural products rises concomitantly, thereby exerting a more pronounced influence on the VOCs of these products. In their study of blueberry drying, Li et al. [83] found that increasing the drying temperature led to an increase in the total number of VOCs contained in dried blueberries. Furthermore, the researchers observed a boost in the amount of VOCs present in fresh blueberries, with the total number rising from 20 to 49, 40, 55, and 51, respectively, at drying temperatures of 50, 60, 70, and 80 °C. A reduction in the drying temperature during the hot air drying process has been observed to positively impact the retention of VOCs in dried agricultural products [84]. In hot air drying, the duration of the drying process is directly correlated with the length of time that physical and chemical reactions within the produce are allowed to occur. Consequently, the quantity of physical and chemical reactions that transpire within the produce increases as the dehydrating time increases, which in turn affects the VOCs. Zhao et al. [85] conducted an investigation into the VOCs of lemons. The findings indicated that the hot air drying yielded 33 and 23 species of VOCs, respectively, after 2 and 4 h. In contrast, the vacuum freeze-drying process yielded 33 and 26 species of VOCs, respectively, after 2 and 12 h. The findings demonstrated that the quantity of VOCs in the lemon decreased as a consequence of an extension of the dehydrating period. In a study of hairtail (*Trichiurus lepturus*) air-drying, Liao et al. [86] identified a notable distinction in the VOCs of hairtail subjected to air drying for 0, 2, and 4 days. The findings specified that the duration of the drying process had a significant effect on the volatile flavor profile of the hairtail. Wang et al. [87] discovered that the VOC content of black tea exhibited notable disparities contingent on the duration of the drying process. Specifically, the flavor quality of black tea subjected to a drying time exceeding 45 min exhibited a pronounced decline. The primary technique for drying agricultural products is hot air drying. The application of hot air enables the production of a variety of VOCs.

### 6.2. Microwave Drying

Microwave drying is a process that employs the use of microwaves to facilitate the removal of water from agricultural products. Ma et al. [88] discovered that uneven microwave drying resulted in localized overheating of *Gastrodia elata*, leading to charring and the production of VOCs (2-ethylfuran, etc.), which resulted in a burnt odor. In contrast, the vacuum freeze-drying process did not result in the same effects. In a study conducted by Dai et al. [89], the drying process of *Sipunculus nudus* was examined. The findings revealed that the total amount of VOCs exhibited a steady rise with the extension of microwave pre-drying time. Compared to the traditional hot air drying technique, the application of microwave pre-drying has been shown to increase the aroma of *Sipunculus nudus*. Lemarcq et al. [90] examined the consequences of microwave roasting on cocoa. Results showed that higher microwave power caused more structural damage, dehydration and other defects. It facilitated the formation of burnt fragrances, the vaporisation of aroma molecules, and oxidation reactions. The Maillard reaction was more pronounced during microwave roasting, resulting in a more intense colour and cocoa aroma in a shortened roasting time. The process of microwave drying is both more rapid and more energy-efficient than traditional drying methods. Additionally, the application of microwave in the drying process imparts a distinctive flavor profile to the produce.

### 6.3. Vacuum Freeze Drying

Freeze-drying is a crucial technique for the preservation of VOCs in agricultural products. It involves freezing the product at sub-zero temperatures, which causes the sublimation of water, thereby removing it from the substance. In a research conducted by Luo et al. [91], the drying of *Clausena anisum-olens* (*Blanco*) was examined. The findings indicated that vacuum freeze-drying was a more effective method than hot air drying for preserving the volatile components and color quality of *Clausena anisum-olens*. In an investigation conducted by Feng et al. [92], a notable distinction was observed in the VOCs of garlic subjected to vacuum freeze drying, when compared to other non-thermal drying techniques, including hot air drying, infrared hot air drying, vacuum radiofrequency drying, and relative humidity drying. In accordance with the OAV > 1 criterion, Huang et al. [93] identified 18 VOCs in freeze-dried coffee leaves, comprising 5 alcohols, 9 aldehydes, 1 olefin, 2 ketones, and 1 ester. In hot-air-dried coffee leaves, 16 VOCs were identified, including 4 alcohols, 7 aldehydes, 4 ketones, and 1 ester. The discrepancies between the two processes may be attributed to the inactivation of enzymes, the loss of aroma precursors, and the generation of novel aroma compounds during hot-air drying. The lowest temperature employed in the freeze-drying process is the most efficacious at impeding the myriad physical and chemical reactions that occur during the drying phase. This process allows the original flavor of agricultural products to be maintained, which is particularly beneficial for products with a high economic value and a rich flavor profile.

### 6.4. Infrared Drying

Infrared drying employs infrared radiation to facilitate the removal of moisture from agricultural products. The research conducted by Li et al. [94] demonstrated that the intensity of the mango aroma diminished after the drying process. Additionally, the aroma shifted from a peel and citrus scent to a fruity, orange peel, and pineapple aroma, with the mango aroma exhibiting the most prominent characteristics after infrared drying at the RS-3 stage, which is a ripening stage. Luan et al. [95] discovered that IR hot air drying of horseradish resulted in the preservation of fewer VOCs relative to other drying methods. This phenomenon was attributed to the uneven temperature distribution resulting from the non-uniform infrared radiation, which led to the destruction of the heat-sensitive aromatic, aldehyde, and alcohol VOCs. Gao et al. [96] discovered that contact ultrasound joint infrared radiation drying could facilitate the hydrolysis of fat and protein in beef, resulting in the release of free amino acids and free fatty acids and an increase in the production of VOCs resulting from lipid oxidation. This process imparted a distinctive flavor profile to air-dried beef, characterized by notes of barbecue, fruit, and grease. The integration of infrared drying into the conventional drying process for agricultural products has the potential to optimise the efficacy of the drying procedure while simultaneously preserving the distinctive flavor profiles of these products.

### 6.5. Other Drying Methods

In addition to the aforementioned drying methods, a plethora of alternative drying techniques are frequently employed in scientific research. Shen et al. [74] discovered that the use of heat resulted in the loss of VOCs in *Oudemansiella raphanipes*. Ultrasound-assisted therapy can decrease the duration of hot air drying and lower the loss of VOCs compared to independent hot air drying. Zhang et al. [97] discovered that the profiles of volatiles present in iron stick yam subjected to different drying methods—namely, electrohydrodynamic drying, hot air drying, and natural drying—appeared to exhibit similarities. Their analysis revealed that the electrohydrodynamic dried yams exhibited a comparatively higher aldehyde content, with aldehydes and nonanal emerging as the most prevalent compounds within their dried products. In their study of dry-cured meats, Li et al. [98] discovered that an optimal rise in temperature is conducive to the production of desirable aromas. The generation of volatile compounds was facilitated by the involvement of free amino acids in the Maillard reaction and microbial activities. Additionally, light has been demonstrated to facilitate the creation of aroma compounds in dry-cured meat. In addition to single drying methods, combined drying techniques are frequently utilised in scientific studies, and VOCs under combined drying techniques are delineated in Table 3. The combined drying method integrates multiple technologies for synergistic operation, achieving energy savings while preserving product quality. The material’s adaptability to diverse substrates has been demonstrated, leading to notable improvements in efficiency and product quality.

## 7. Conclusions

The drying process of agricultural products is distinguished by a plethora of physical and chemical reactions, with the Maillard reaction and lipid oxidation reaction occupying a primary position, while the enzymatic reaction and fermentation reaction assume a secondary role. The collective impact of these physicochemical reactions endows the produce with a distinctive flavor profile. The most appropriate methodologies for the determination of these VOCs are E-nose, GC-MS, and GC-IMS. E-nose is frequently employed to discern overall flavor discrepancies between samples, whereas GC-MS and GC-IMS exhibit varying sensitivities for diverse classes of compounds present in agricultural products. In practice, the three assays are often employed in conjunction to ensure the comprehensive retention of information pertaining to the volatile components of produce. By employing mathematical techniques and incorporating human olfactory perceptions, the VOCs present in dried agricultural products can be evaluated through the use of SA and OAV. The impact of various drying techniques on VOCs in agricultural products is variable. The greatest detrimental effect on these substances is observed in the case of hot air drying. In comparison to diverse heat treatment drying techniques, vacuum freeze-drying has been demonstrated to result in superior retention of flavor substances in agricultural products. This article contributes to a more profound understanding of VOCs following the drying of agricultural products, and provides a theoretical foundation for subsequent developments and applications in the utilization of agricultural resources and related research.

## Figures and Tables

**Table 2:** Sensor array of PEN3.0 electronic nose and its main properties.

Sensor No.	Sensor Name	Description of Performance
S1	W1C	Aromatic compounds
S2	W5S	Nitrogenous compounds
S3	W3C	Ammonia, Aromatic compounds
S4	W6S	Hydrocarbons
S5	W5C	Alkanes, Olefins, Aromatic compounds
S6	W1S	Alkanes
S7	W1W	Sulfur-containing compounds
S8	W2S	Alcohols, Some Aromatic Compounds
S9	W2W	Aromatic compounds, organosulfur compounds
S10	W3S	Alkanes, Aliphatics

**Table 3:** Research on combined drying methods for agricultural products.

No.	Drying Method	Appropriate Agricultural Products
1	Ultraviolet-assisted cold-air drying	Pacific saury (*Cololabis saira*) [23]
2	Vacuum-infrared drying	Orange peels [99]
3	Hot air drying combined with baking	Whole lotus root powders [100]
4	Infrared-hot air drying	Chrysanthemum (*Chrysanthemum morifolium* Ramat.) [101]
5	Far infrared-hot air drying	*Anoectochilus* [102]

## Data Availability

No new data were created or analyzed in this study. Data sharing is not applicable.
